# Computerized Versus Traditional Approaches for Total Knee Arthroplasty: A Quantitative Analysis of Knee Society Score and Western Ontario and McMaster Universities Osteoarthritis Index

**DOI:** 10.1111/os.14103

**Published:** 2024-05-26

**Authors:** Srikar R Namireddy, Saran S Gill, Yousuf Yaqub, Pratik Ramkumar

**Affiliations:** ^1^ Faculty of Medicine, Imperial College London London UK

**Keywords:** Computerized TKA, KSS, Traditional TKA, WOMAC

## Abstract

Total knee arthroplasty (TKA) is a common surgery for osteoarthritis, with increasing prevalence expected in the near future. This systematic review and meta‐analysis compared the effectiveness of computerized TKA versus traditional TKA, focusing on postoperative outcomes measured by the Western Ontario and McMaster Universities osteoarthritis index (WOMAC) and the Knee Society score (KSS). A search on PubMed and Cochrane databases on November 14, 2023 for retrospective randomized controlled trials (RCTs) yielded data on WOMAC and KSS. The search strategy was predefined, and methodological quality of studies was critically appraised. Two researchers extracted data. Unpaired *t*‐testing assessed the mean monthly changes in KSS and WOMAC for computer‐aided versus traditional TKA. Review Manager 5.3 was used for data synthesis and analysis. Out of 729 records, five RCTs enrolling 339 patients were eligible and analyzed using a random effects meta‐analysis. The mean monthly ΔKSS score differed significantly between the traditional and computerized groups (11.47 ± 8.76 vs. 9.26 ± 6.05, respectively; *p* < 0.01). However, the pooled mean difference estimate showed no significant differences (*D* = 0.20, 95% CI = −0.53 to 0.93, *p* = 0.59), with high heterogeneity (*I* ^2^ = 85%, *p* < 0.001). The mean monthly ΔWOMAC score also differed significantly (−14.18 ± 21.54 vs. −18.43 ± 20.65, respectively; *p* < 0.05), but again, no significant differences were found in the pooled estimate (*D* = 0.17, 95% CI = −0.46 to 0.79, *p* = 0.60), with moderate heterogeneity (*I* ^2^ = 28%, *p* = 0.24).There is no significant difference in KSS or WOMAC outcomes between traditional and computerized TKA. The study suggests the need for further research with longer follow‐up periods, more timepoints, and a broader range of patient outcome measures to fully evaluate the advantages and disadvantages of each method.

## Graphical abstract

There is no significant difference in McMaster Universities osteoarthritis index (WOMAC) and the Knee Society score (KSS) outcomes between traditional and computerized total knee arthroplasty (TKA). The study suggests the need for further research with longer follow‐up periods, more timepoints, and a broader range of patient outcome measures to fully evaluate the advantages and disadvantages of each method.

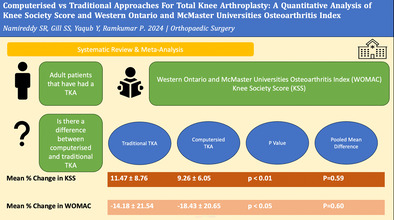

## Background/Introduction

Total knee arthroplasty, or replacement, (TKA) is a complete reconstruction of the knee joint.^1^ This often occurs after osteoarthritis (OA) of the knee, in two or more compartments.^1^ With over 365 million cases of knee OA globally,^2^ the TKA is considered a cost effective intervention to improve quality of life, function and pain.^3^ There has been an increase in annual TKA procedures being performed,^4^ with projections of a 673% increase by 2030 in the United States,^5^ and as such methods should evolve with the demand. Studies have reported that computer aided TKA, when compared to traditional TKA methods, has been shown to have differing outcomes.^6^ Therefore highlighting the need for more research on the topic.

The Western Ontario and McMaster Universities osteoarthritis index (WOMAC) is a global outcome instrument that measures three domains: pain, function and stiffness. It consists of 24 questions in the form of a likert scale format^7^ allowing for a nuanced assessment of a patient's condition and response to treatment. The WOMAC score is particularly valuable in the context of osteoarthritis, as it provides a comprehensive evaluation of the areas most impacted by disease. The Knee Society score (KSS) is another instrument used to assess outcomes. It is designed to assess not only the clinical aspects of the knee, such as range of motion and stability, but also the patient's functional abilities (i.e., walking and climbing stairs). The KSS is divided into two distinct parts:^8^ one that evaluates the knee itself (the knee score) and another that assesses the patient's functional capabilities (the function score). This dual approach enables a holistic understanding of the surgical outcomes, making the KSS an invaluable tool in the context of TKA.

This study intends to assess the literature to come to a quantitative conclusion to assess the superiority, if any, of traditional TKA compared to computer aided TKA, by working out rate of change between baseline and time periods post operatively for both surgical techniques. This will be done *via* pooling data to perform a “random effects” meta analysis, using WOMAC and KSS as proxies.

## Methods

### Literature Search

This systematic review and meta‐analysis was performed in conjunction with the PRISMA (Preferred Reporting Items for Systematic reviews and Meta‐analyses) statement. Articles were identified *via* the PubMed database and the Cochrane Library. The Cochrane Library sourced papers from: Embase, PubMed, CT.gov and ICTRP. This search was completed on November 14 2023. The PubMed search used both MESH terms and free terms for: (Surgery OR (Robotic* OR Computer OR Robot*)) AND (TKA OR Total Knee Replacement OR Total Knee Arthroplasty OR TKA) AND ((WOMAC OR The Western Ontario and McMaster Universities Osteoarthritis Index) AND (KSS OR Knee Society Score)). The search of the Cochrane Library also used MESH terms and free terms. “Total knee arthroplasty” (In the abstract, keywords or title) AND “computer aided” AND WOMAC AND KSS AND Surgery formed the search string. All terms bar “total knee arthroplasty” were searched for in the full text, using the advanced search tool.

### Eligibility Criteria

**FIGURE 1:**
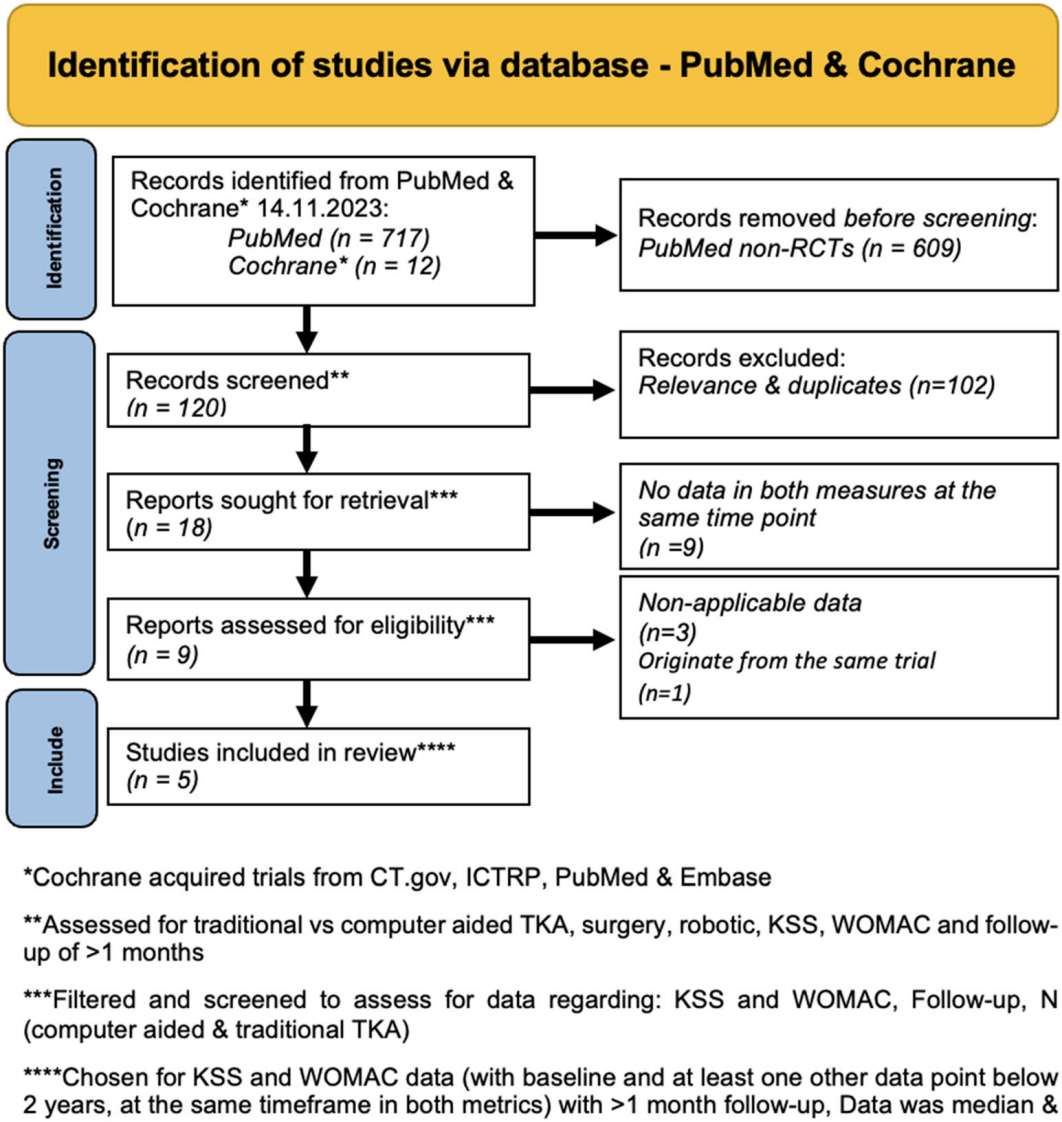
PRISMA diagram describing the process of the literature search.

RCTs, reporting both KSS and WOMAC. Figure 1 describes the screening process. Data extraction was retrieved and reviewed by two of the researchers independently. Any disagreements were solved by general consensus from the remaining authors. The protocol was registered on PROSPERO:CRD42023494031.^9^

### Inclusion Criteria

1. Level 1 evidence.
2. Comparison of both computer aided and traditional TKA methods.
3. Data on both KSS and WOMAC.
4. >1 month of followup.
5. >15 patients per arm of each trial.

### Exclusion Criteria

1. Level II evidence and below.
2. Articles that were not in English.
3. Papers with no baseline data.

### Outcome Measures

Primary outcome: Comparison of % change in mean change in KSS in both computer aided, and traditional, TKA. Secondary outcome: Comparison of % change in mean change in WOMAC in both computer aided, and traditional, TKA. Data was extracted at all available timeframes at 12 months from the TKA and earlier.

### Data Extraction

Data was extracted from each included RCT into a spreadsheet. Data from the baseline, other data points and the N of the arms of each included trial were extracted. Extraction was completed by two researchers on the team, independently. Data was extracted at available timeframes between 12 months from the TKA and earlier.

### Data Synthesis and Statistical Analysis

Both of the outcome measures consisted of continuous data. A mean change, per month, in both KSS and WOMAC was calculated from the data extracted from both the computer guided TKA and traditional TKA arms of included trials. A random effects model was used to perform the meta‐analysis on R software. Results and confidence intervals were displayed in the form of forest plots. The percentage of variation within the studies that can be attributed to heterogeneity was determined using the *I* ^2^ function. Low, moderate and high heterogeneity are defined as <25%, 25%–75% and >75% respectively. Level I data was used in this meta‐analysis.

### Methodological Quality Assessment

All included studies were independently reviewed in relation to quality by two of the authors Any differences were decided by a general consensus between all authors. The risk of bias in each included trials was assessed using the RoB‐2 tool *via* its seven domains, and was scored as: “low” “unclear” and “high” risk:

1. Randomization method.
2. Allocation concealment.
3. Blinding of participants and personnel.
4. Blinding of outcome assessment.
5. Incomplete outcome measures.
6. Selective reporting bias.
7. “Other” forms of bias.

### Publication Bias

Publication bias was not evaluated, since only five articles were included in this systematic review and meta‐analysis.

## Results

### Literature Search Results

The search in the PubMed database yielded 717 results. When filtered down to RCTs there were 108 articles. The search in the Cochrane Library consisted of 12 trials. Overall, five studies were included, encompassing 339 participants. All five studies were included in the meta‐analysis. Figure 1 shows a PRISMA diagram of this search.

### Quality Assessment

**FIGURE 2:**
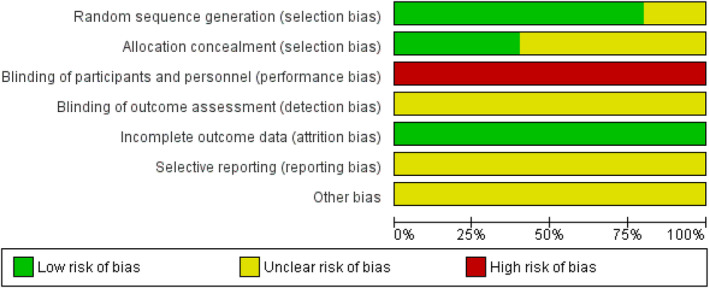
A diagrammatic representation of the ROB2 assessment performed for each of the included randomized studies. The color represents the quality in each of the domains (Red = high risk, Yellow = unclear risk, Green = low risk).

The majority of studies described suitable random sequence generation and randomization methods. However, there were variable levels of bias due to the methods of allocation concealment across the RCT's considered. All studies were affected by high risk of bias for the blinding of both participants and personnel, as highlighted in Figure 2. This was partly due to the inherent inability to blind the operative team. The risk of bias assessment across all the randomized controlled trials of included studies is displayed in Table 1.

**TABLE 1:** A table displaying the risk of bias for each of the included randomized studies.

	Random sequence generation (selection bias)	Allocation concealment (selection bias)	Blinding of participants and personnel (performance bias)	Blinding of outcome assessment (detection bias)	Incomplete outcome data (attrition bias)	Selective reporting (reporting bias)	Other bias
Decking *et al*. 2007^10^	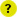	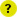	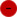	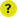	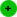	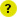	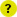
Kotela *et al*. 2015^11^	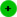	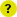	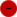	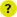	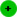	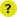	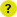
Spencer *et al*. 2007^12^	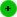	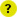	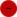	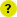	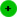	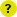	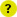
Xu *et al*. 2022^13^	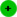	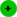	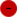	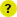	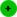	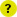	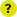
Lüring *et al*. 2008^14^	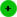	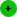	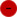	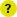	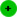	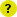	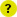

*Note*: The color represents the quality in each of the domains (Red = high risk, Yellow = uncertain, Green = low risk).

### Characteristics of Studies Included

The details of the five studies (five RCTs) included in the systematic review are summarized in Table 2

**TABLE 2:** Characteristics of publications included in the present study.

Author	Journal	Location	TTKA Method	TTKA n	CTKA Method	CTKA n	Form of data		Data used
							WOMAC	KSS	
Xu *et al*.^13^	Orthopedic Surgery	China	Conventional manual total knee arthroplasty	35	Domestic robot‐assisted total knee arthroplasty	37	Mean & SD	Median & IQR	Baseline, 90 days
Spencer *et al*.^12^	Bone & Joint Journal	Australia	Conventional jig based	30	Computer navigated	30	Mean & SD	Mean & SD	Baseline, 1 year
Lüring *et al*.^14^	Knee Surgery, Sports Traumatology, Arthroscopy	Germany	Minimally invasive surgery	30	Computer assisted surgery	30	Mean & SD	Mean & SD	Baseline, 12 weeks
Decking *et al*.^10^	Acta ChirurgiaeOrthopaedicae Et TraumatologiaeČechoslovaca	Germany	Conventional (manual)	25	Computer navigated	27	N/A	Mean & SD	Baseline, 12 months
Kotela a *et al*.^11^	BioMed Research International	Poland	Conventional instrumentation	46	Signature CT‐based implant positioning system	49	Mean & SD	Mean & SD	Baseline, 1 year

Abbreviations: CTKA, Computer aided TKA; TTKA, Traditional TKA.

### Primary Outcome: Knee Society Score (KSS)

**FIGURE 3:**
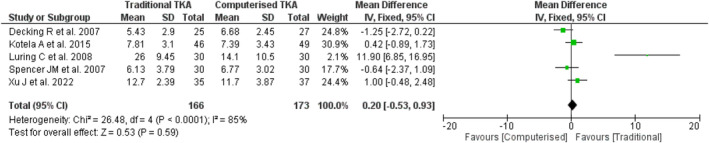
Forest plot comparing the monthly rate of change in KSS between the traditional TKA group and the computerized TKA group.

Five studies^10^, ^11^, ^12^, ^13^, ^14^ included data to perform the systematic review and meta analysis for monthly rate of ΔKSS score, measured using a 0 to 100 scale, across 10 domains. The mean monthly rate of ΔKSS score differed significantly between traditional and computerized groups (11.47 ± 8.76 vs. 9.26 ± 6.05, respectively; *p* < 0.01). When considering the effect size, the overall pooled mean difference estimate showed no significant difference in monthly rate of ΔKSS between traditional and computerized groups (*D* = 0.20, 95% CI = −0.53 to 0.93, *p* = 0.59), with high heterogeneity (*I* ^2^ = 85%, *p* < 0.001) (Figure 3).

### Secondary Outcome: Western Ontario and McMaster Universities Osteoarthritis Index (WOMAC)

**FIGURE 4:**
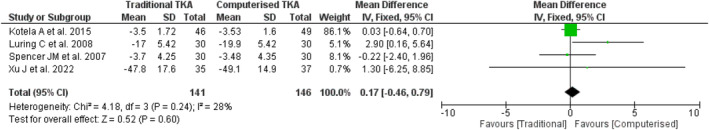
Forest plot comparing the monthly rate of change in WOMAC score between the traditional TKA group and the computerized TKA group.

Four studies^11^, ^12^, ^13^, ^14^ included data to perform the systematic review and meta analysis for monthly rate of ΔWOMAC score, measured using a 24‐item questionnaire, spanning three modules. The mean monthly rate of ΔWOMAC score differed significantly between traditional and computerized groups (−14.18 ± 21.54 vs. −18.43 ± 20.65, respectively; *p* < 0.05). When considering the effect size, the overall pooled mean difference estimate showed no significant difference in monthly rate of ΔKSS between traditional and computerized groups (*D* = 0.17, 95% CI = −0.46 to 0.79, *p* = 0.60), with moderate heterogeneity (*I* ^2^ = 28%, *p* = 0.24) (Figure 4).

## Discussion

The evidence in this study is derived from five peer‐reviewed and published randomized control trials, comparing differences in percentage change in 1 year outcomes after computer aided versus traditional TKA. This review attempted to quantitatively analyze and assess differences in WOMAC and KSS, irrespective of sex, age and demographic factors. The studies analyzed came from China,^13^ Australia,^12^ Germany,^10^, ^14^ and Poland.^11^ All five studies made use of KSS to measure patient outcomes after 1 year, and all studies also made use of WOMAC to measure patient outcomes after 1 year. Selected studies covered a large geographical area. Each paper followed an intention‐to‐treat analysis when considering both KSS and WOMAC.

### Study Analysis

Xu *et al*.'s paper from China^13^ was a randomized control study assessing early clinical and radiographic outcomes of robot‐assisted total knee arthroplasty (RA‐TKA) in comparison with conventional manual TKA (CM‐TKA) techniques. The study prospectively enrolled 77 patients, of which 72 were selected to participate in the trial, all of which fell within the age range of 18–80 years old. This was a relatively small sample size, which we considered when conducting our meta‐analysis. Patients were then randomly divided into a RA‐TKA group (*n* = 37) and a CM‐TKA group (*n* = 35). Improvements in knee function were assessed *via* knee range of motion (ROM), WOMAC and KSS. This study concluded that while both groups saw significant improvements in WOMAC and KSS scores, there were no significant differences in either group for both assessments. This mirrored our own findings, where there were no significant differences in ΔKSS and ΔWOMAC on a monthly basis. A concern with this paper was the relatively short follow‐up period of 90 days, highlighting a need for long term efficacy in terms of post‐operative functional scores to be further analyzed. However, this paper was published in 2022, and therefore considered to be up to date with current data, and as such made this paper viable for inclusion in our analysis.

Spencer *et al*.'s Australian study^12^ was also a randomized control trial. Seventy‐two patients were randomly allocated to undergo computer‐navigated or conventional replacement, of which only 60 patients were available for assessment *via* KSS, the short form‐36 health survey, WOMAC, the Bartlett patellar pain questionnaire and the Oxford knee score, none of which had undergone any form of revision. Similar to the paper from China^13^ and our own findings, none of the measures of post‐operative outcome showed any significant differences, despite better alignment achieved by computer‐navigated surgery.

Lüring *et al*.'s^10^ paper was a prospective randomized trial based in Germany involving 90 patients who were randomized into three groups of 30 patients: computer assisted surgery‐Minimally invasive surgery (CAS‐MIS), freehand minimally invasive surgery (freehand MIS), and a freehand conventional group. All three groups were similar regarding patient parameters. Initially the study hoped to investigate all patients at 1, 6 and 12 weeks, 6 and 12 months after the operation. However as there were no statistical differences in KSS and WOMAC by the 12‐week mark between the three groups, follow up was terminated. All patients fell within the 60–80 age range. One major limitation of this paper as described above was the relatively short follow‐up time in the study. While there were no significant differences in WOMAC or KSS at any of the time points in any of the groups, similar to Xu *et al*.'s paper,^13^ a lack of long‐term follow‐up highlights the need for randomized control trials assessing longer‐term post‐operative outcomes. Overall, all four researchers and Luring *et al*. considered the results to be valid, and as such the paper was included in our analysis.

Decking *et al*.'s paper also from Germany, was again, a randomized control trial^10^ After ruling out patients *via* their exclusion criteria, 52 patients were randomized, with one group to receive a conventional total knee replacement and one group to receive a total knee replacement assisted by kinematic computer‐navigation (*n* = 25, *n* = 27 respectively) (OrthoPilot System, Software 2.2, Aesculap, Tuttlingen, Germany). Patients started physical therapy on the day after the operation, all patients were limited to partial weight bearing until wound healing was secured. At the 3 month follow‐up, the mechanical alignment of the leg as measured on full‐leg, weight bearing, standing radiographs, had reached a straight axis in more cases with the computer navigated implantation compared to the conventional implantations. This difference was statistically significant. However at the 12 month follow‐up, when KSS and WOMAC were assessed, no statistically significant differences in ΔKSS and ΔWOMAC could be observed between both groups. A significant limitation of this study was the small sample size. Decking *et al*. calculated the minimum sample size required to show a significant difference in “physical function” using power analysis, and proposed that 1102 randomized patients would be needed in the follow‐up in order to observe and discernible differences in outcomes for the two procedures.

Finally, Kotela *et al*.'s^11^ study from Poland, also conducted a prospective, randomized control trial which showed similar results, with no significant difference in post‐operative assessments in both Patient‐specific CT based instrumentation (PSI) and Conventional instrumentation groups (*p* = 0.07, *p* = 0.92, KSS and WOMAC respectively). This study consisted of 112 patients split into the two aforementioned groups (*n* = 62, *n* = 50, PSI and CI respectively). While these results are consistent with our finding as well the other aforementioned papers, it is worth noting that PSI is a markedly different procedure from the others used in the previous studies. This may have introduced some level of homogeneity to our analysis, however due to the long follow‐up time of the study, as well as all four researchers considering the results to be valid, it was included as part of our paper.

### Wider Context

Our study findings align with those of several other relevant studies, which collectively suggest that there is no statistically significant difference in post‐operative outcomes between traditional manual TKA and computer‐assisted techniques.^10^, ^11^, ^12^, ^13^ Additionally, in a randomized control trial by Kim *et al*,^15^ it was demonstrated that there was no significant differences in functional outcome scores, aseptic loosening, overall survivorship, and complications between robotic and traditional TKA, further remarking that due to additional time and cost investment, they could not recommend robotic intervention over traditional measures.

Despite variations in methodologies and patient populations across these studies, the consistent lack of significant differences in measures such as Knee Society score (KSS) and Western Ontario and McMaster Universities osteoarthritis index (WOMAC) scores underpins the importance of our findings. While computer‐assisted techniques may offer advantages in terms of alignment precision and intraoperative decision‐making,^16^ our results suggest that these benefits may not translate into superior functional outcomes or patient‐reported satisfaction in the short to medium term. However, it is essential to acknowledge the limitations inherent in these studies, such as relatively short follow‐up periods and variations in sample sizes. Further research, including longer‐term follow‐up and larger‐scale randomized controlled trials, is warranted to provide more definitive conclusions regarding the comparative effectiveness of traditional and computerized TKA techniques.

### Limitations and Strengths

The variability in study designs, patient populations and follow up periods among the included studies introduces a considerable degree of heterogeneity that may affect the generalizability of the results. The varying sample sizes of the studies in particular is a significant limitation, with certain studies having relatively small cohorts—none of the arms of any of the included papers consist of more than 50 patients. This could compromise the statistical power of the findings while introducing an element of sampling bias. Similarly, the varying availability of data, meant that we had to decide on a 2 year cut‐off for data usage, as we could not effectively compare a change in WOMAC or KSS per month between 0 and 2 years post‐operation, to that of a change from 0 to 15 years post‐operation, for example. In addition to this, while the studies included in the review are geographically diverse there may be other unmeasured cultural or healthcare system related factors that could influence the outcomes which have not been accounted for in this analysis. Finally, the reliance on only two outcome measures (WOMAC and KSS) may have omitted the consideration of certain factors relevant to patient recovery post TKA, which might have been captured by a more holistic approach. Despite these limitations, we believe that this review offers a valuable insight into the differences in recovery between traditional and computerized TKA, and will hopefully serve as a foundation for future research within this field.

## Conclusion

Overall, the analysis reveals no statistically significant difference in the monthly rate of change in KSS and WOMAC scores between traditional and computerized TKA groups. This would suggest that (in terms of patient outcomes captured by KSS and WOMAC) both computerized and traditional TKA methods are comparatively effective. In order to gain a more complete picture of the advantage of one method over the other in terms of long‐term patient outcomes, further research with longer follow up periods, more frequent data collection, and a more comprehensive/diverse range of outcome measures is needed.

## Conflict of Interest Statement

The authors certify that they have no affiliations with or involvement in any organization or entity with any financial interest (such as honoraria; educational grants; participation in speakers' bureaus; membership, employment, consultancies, stock ownership, or other equity interest; and expert testimony or patent‐licensing arrangements), or non‐financial interest (such as personal or professional relationships, affiliations, knowledge or beliefs) in the subject matter or materials discussed in this manuscript.

## Author Contributions

All authors have contributed equally to this paper. SRN has carried out the majority of statistical analysis. SSG undertook the literature search, data collection and statistical analysis, assisted by SRN. YY and PR were involved in the write‐up and editing process. All authors have read and approved the final submitted manuscript. SRN and SSG share first author status.

## Data Availability

All data generated or analyzed during this study are included in this published article.
